# Feature-based volumetric defect classification in metal additive manufacturing

**DOI:** 10.1038/s41467-022-34122-x

**Published:** 2022-10-26

**Authors:** Arun Poudel, Mohammad Salman Yasin, Jiafeng Ye, Jia Liu, Aleksandr Vinel, Shuai Shao, Nima Shamsaei

**Affiliations:** 1 National Center for Additive Manufacturing Excellence (NCAME), Auburn University, Auburn, AL 36849 USA; 2 Department of Mechanical Engineering, Auburn University, Auburn, AL 36849 USA; 3 Department of Industrial and Systems Engineering, Auburn University, Auburn, AL 36849 USA

**Keywords:** Mechanical engineering, Statistics

## Abstract

Volumetric defect types commonly observed in the additively manufactured parts differ in their morphologies ascribed to their formation mechanisms. Using high-resolution X-ray computed tomography, this study analyzes the morphological features of volumetric defects, and their statistical distribution, in laser powder bed fused Ti-6Al-4V. The geometries of three common types of volumetric defects; i.e., lack of fusions, gas-entrapped pores, and keyholes, are quantified by nine parameters including maximum dimension, roundness, sparseness, aspect ratio, and more. It is shown that the three defect types share overlaps of different degrees in the ranges of their morphological parameters; thus, employing only one or two parameters cannot uniquely determine a defect’s type. To overcome this challenge, a defect classification methodology incorporating multiple morphological parameters has been proposed. In this work, by employing the most discriminating parameters, this methodology has been shown effective when implemented into decision tree (>98% accuracy) and artificial neural network (>99% accuracy).

## Abstract (web summary)

Additively manufactured materials contain different types of volumetric defects. Here, the authors utilize the most distinguishing morphological features among different defect types to propose a defect classification methodology.

## Introduction

**Fig. 1:**
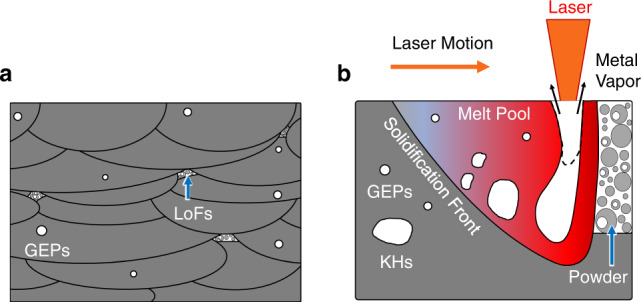
Schematic illustrations of defects formation during L-PBF. **a** Formation of LoFs and GEPs. **b** Formation of KHs and GEPs.

Additive manufacturing (AM) technologies such as laser powder bed fusion (L-PBF), with its layer-by-layer fabrication strategy and flexible feedstock, offer advantages over conventional manufacturing and can fabricate complex-shaped parts, consolidate assemblies into integral components, reduce lead time, and manufacture in remote locations^1,2^. However, AM parts are also prone to be laden with volumetric defects^2^. In the machined condition, these defects—commonly lack of fusions (LoFs), gas-entrapped pores (GEPs), and keyholes (KHs)—act as stress risers and are detrimental to the mechanical, especially fatigue, properties of AM parts^3–6^. For L-PBF processes, LoFs mainly form due to insufficient overlap between adjacent melt pools, between layers or laser tracks^7,8^, which can be the result of insufficient energy input or excessively large hatch distance (see Fig. 1(a)). KHs form in overheating conditions due to the “pinch-off” from the bottom of the depression inside the melt pool^9,10^ (see Fig. 1(b)). On the other hand, GEPs are essentially “bubbles” of inert gas inside/between powder particles entrapped within the melt pool due to the combined action of buoyancy, Marangoni force, the turbulence due to vapor recoil, and the rapidly moving solidification front^11^ (see Fig. 1(a & b)). GEPs cannot be avoided and are typically present even under optimum processing conditions^12,13^.

For L-PBF processes, these volumetric defects contain shielding gases (such as argon (Ar)), which are often insoluble to metal. Therefore, the removal of the defects is challenging, if not impossible, since it would require the complete expulsion of such gases through the bulk of the metal. For instance, the author’s recent work had shown that Ar is insoluble in titanium and, during hot isostatic pressing (HIP), back pressure builds up within the defects as they shrink which counteracts the pressure of the working medium and prevents full defect closure^14–16^. Indeed, driven by the internal pressure, defects had been observed to regrow during the post-HIP heat treatment^17,18^. Therefore, compared to remedial efforts, optimizing the AM process parameters to minimize both the size and population of defects during fabrication is perhaps a more crucial step to ensure the structural integrity of AM parts^19^.

Process optimization for a new material can be labor-intensive and require the fabrication/characterization of a large number of microstructural coupons with incrementing process parameters to map the print quality of a parameter space. For instance, only 5 variations of 3 process parameters (such as laser power, laser speed, and lahatch dist for L-PBF) produce 125 coupons. The coupled nature between processing conditions and the classification of volumetric defects^20^ can help expedite the procedure by indicating the print quality as well as the course of correction. For instance, the presence of either LoFs or KHs suggests that the process parameters are non-optimal. Removing LoFs requires increasing energy input (increasing laser power and/or reducing laser speed) and/or reducing layer thickness/hatch distance. To remove KHs, energy input would need to be reduced. Conversely, the accurate classification of defects can also enable effective seeding of desired defects in material to understand the critical effects of volumetric defects on fatigue behavior. Historically, information on defects has been two-dimensional (2D) and is obtained by polished-section microscopy. Recently, the widespread adoption of X-ray computed tomography (XCT) systems permitted three-dimensional (3D) characterization of volumetric defects which facilitates their more reliable classification.

**Fig. 2:**
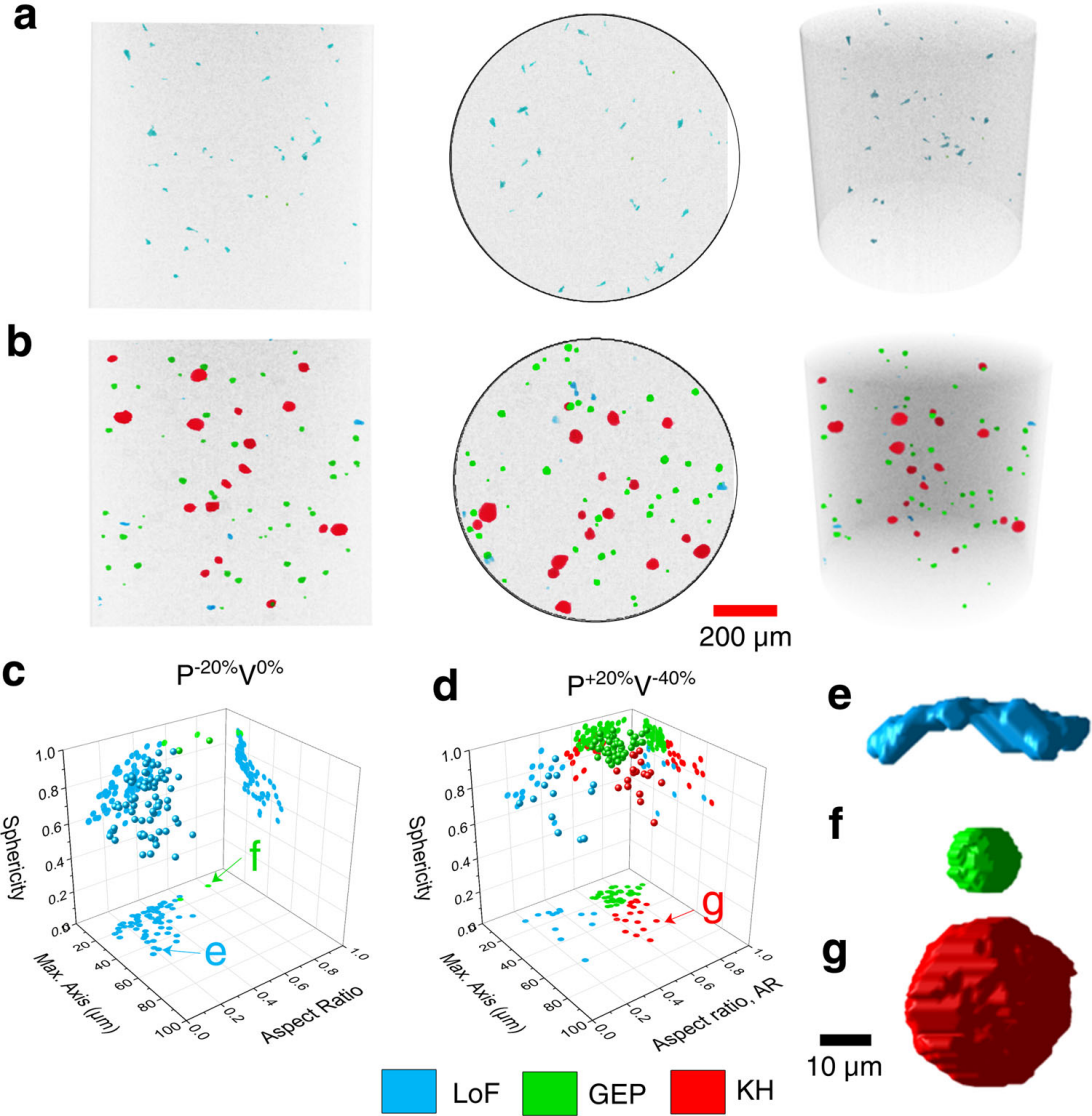
Visualization of volumetric defects in L-PBF coupons. Front, top, and isometric views of the XCT scan region of coupons fabricated using **a** P^−20%^V^0%^ and **b** P^+20%^V^−40%^ parameters. Scatterplots in the 3D space of size, aspect ratio, and sphericity showing the volumetric defects observed in **c** P^−20%^V^0%^ and **d** P^+20%^V^−40%^ coupons. Appearances of selected defects, **e** a LoF, **f** a GEP, and **g** a KH are also shown.

For defect classification, although limited attempts with machine learning (ML)—such as K-means clustering^21^—have been made, the most common approach is perhaps by setting limits on morphological parameters such as size, sphericity, and aspect ratio^21–24^. For a defect, its sphericity is the surface area of an equal-volume sphere divided by that of itself; and aspect ratio is the ratio of its smallest to its largest orthogonal dimensions. Owing to their origins, the sphericity and the aspect ratio of the LoFs (see Fig. 2(e)) are typically the lowest^21,22,25^, followed by those of the KHs (see Fig. 2(g))^22^ and GEPs (see Fig. 2(f))^23,25,26^. As for size, LoFs can be either very large (up to a few millimeters between layers) or very small (down to a few micrometers between molten tracks). In contrast, KHs observed in L-PBF parts are typically smaller than 100 µm (i.e., dependent on the laser spot diameter) and GEPs are even smaller. Even though the formation mechanisms of various types of defects are well known^9,27–29^, classifying them according to one or two simple measures of size, sphericity, and aspect ratio has been challenging. This could be explained by different defect types sharing overlaps in their value ranges of these parameters; therefore, they cannot be distinguished by a clear threshold. In addition, due to differences in defect characteristics resulting from different fabrication platforms, process parameters, and XCT scanning parameters, discrepancy exists among existing criteria (see “Results” section for the complete evaluation of existing classification criteria)^9,21–26^.

This work endeavors to devise a robust approach to classify the volumetric defects and identify the most distinguishing features among them using L-PBF, as the most commonly utilized AM process, and Ti-6Al-4V, as a material with a lot of applications in aviation, space, and medical fields^30^. A large quantity of Ti-6Al-4V coupons were fabricated by L-PBF with varying process parameters (laser power (P), laser speed (V), and hatch distance (H)) to induce different types of volumetric defects. The coupons were then XCT scanned and the data post-processed to inspect defects and characterize them with a total of nine morphological parameters. A total of 1970 defects were manually labeled, their morphological parameters calculated, and the statistics of the morphological features analyzed. It was shown that overlaps of morphological parameters always existed in their value ranges among different defect types and using multiple parameters could reduce the probability of a defect falling on multiple overlaps. Implementing this philosophy, decision tree- and artificial neural network-based defect classification schemes utilizing multiple morphological parameters have been demonstrated with success.

## Results

### Evaluation of existing classification criteria

Although ML methods such as K-means clustering^21^ have been used, the most common approaches for defect classification are still based on thresholds set on one or two parameters among size (such as the maximum dimension), sphericity, and aspect ratio^21–25^. The existing limit-based criteria in the literature for distinguishing LoF, GEP, and KH defects based on XCT scans are summarized in Table 1. The listed criteria have all been established for Ti-6Al-4V fabricated by L-PBF processes. However, the defects analyzed in these studies were induced from different L-PBF systems, fabricated with different process parameters, and/or XCT scanned at different voxel sizes, which likely have caused some variations from one another even for the same defect types. For instance, as shown in Table 1, although Snell et al., Kasperovich et al., and Vilaro et al. have all specified that LoFs should be larger than 100 µm, Snell et al. and Kasperovich et al. also stipulated thresholds for sphericity. LoFs are defined to have sphericity lower than 0.6 and 0.7 by Snell et al. and Kasperovich et al., respectively. In addition, Snell et al. and Kasperovich et al. have proposed KHs to, respectively, be greater than 100 µm and 50 µm, with the former based on du Plessis et al.^31^. One of the reasons for the difference in the size threshold for KHs might have been the use of different voxel sizes during XCT scans (see Table 1 for the voxel sizes used in these studies). The use of smaller voxel size can result in the detection of smaller-sized defects^32^.

**Table 1:** Classification criteria for defects in L-PBF Ti-6Al-4V available in the literature proposed based on XCT scans of different voxel sizes. S, AR, and Sph. represent the size, aspect ratio, and sphericity of the defects, respectively

Study	Voxel Size (µm)	LoF			GEP			KH		
		S (µm)	AR	Sph.	S (µm)	AR	Sph.	S (µm)	AR	Sph.
Snell^21^	8.9	> 100	–	<0.6	≤100^a^	≥0.5^a^	≥0.6^a^	> 100	<0.5	–
Kasperovich^22^	0.3	> 100	–	<0.7	≤50^a^	–	>0.7^a^	> 50	–	> 0.7
Vilaro^23^	N/A	>100	–	–	–	–	–	–	–	–
P^−20%^V^0%^ (This work)	1	10.1–47.8	0.1–0.6	0.5–0.9	10.6–13.2	0.6–0.8	0.8–0.9	–	–	–
P^+20%^V^−40%^ (This work)	1	10.5–59.9	0.2–0.5	0.5–0.9	11.1–28.5	0.6–0.9	0.8–1.0	30.3–65.8	0.6–0.8	0.7–0.9

^a^Criteria for GEPs were not defined by Snell et al. and Kasperovich et al. Rather, they were implied from the criteria for LoFs and KHs.

The efficacy of the classification criteria shown in Table 1 has been evaluated in this study using manually labeled (see “Methods” for labeling procedure) defects contained in coupons fabricated with P^−20%^V^0%^ (i.e., laser power decreased by 20% from manufacturer recommended parameters, which are provided in “Methods section”) and P^+20%^V^−40%^ (i.e., laser power increased by 20% and laser speed decreased by 40% from manufacturer recommendation) parameters. The P^−20%^V^0%^ and P^+20%^V^−40%^ parameters respectively correspond to overall underheating and overheating conditions and, therefore, are expected to induce LoFs and KHs. The GEPs are essentially intrinsic to the L-PBF process and are expected to be present in both conditions. Some LoFs may also form in the overheating condition due to the highly turbulent melt pools and the resulting highly non-uniform molten track cross-sections. Indeed as shown in Fig. 2(a, b), manual defect labeling, agnostic of the processing conditions of each coupon, has confirmed the presence of only LoFs and GEPs in the underheating condition and all three defect types in the overheating condition.

In addition, the P^+20%^V^−40%^ coupon also contains significantly more GEPs compared to the P^−20%^V^0%^ one, which may be ascribed to the more severe melt pool turbulence in the overheating condition that may have hindered GEPs’ escape. It is also possible that very small KHs exist^33^ in the P^+20%^V^−40%^ coupon; however, limited by the XCT’s resolution, they may be mislabeled as GEPs. Nevertheless, within this size range (i.e., <30 µm) the effects of KHs and GEPs on fatigue behavior are similar; the mislabeling is therefore not consequential. The size, sphericity, and aspect ratio of LoFs, GEPs, and KHs in these two coupons are presented in 3D space in Fig. 2(c, d). From the labeled data, the ranges of the three parameters for each defect type are reported in Table 1 as well as shown in Supplementary Fig. 1(a–f) for comparison. Visual comparisons of the classification criteria listed in Table 1 are also provided in Supplementary Fig. 2.

It is evident that the direct application of these criteria cannot fully classify the defects within the two coupons, which is likely due to the aforementioned differences in L-PBF process and XCT scanning parameters. More importantly, it is noted from Table 1 that the three defect types observed in the P^−20%^V^0%^ and P^+20%^V^−40%^ coupons had overlaps of various degrees in the ranges of all three morphological parameters. Therefore, the popular approach of setting limits on one or two of these parameters may not be sufficient to determine the defect types. Therefore, the simultaneous usage of a few most discriminating parameters may be needed.

### Statistics on morphological parameters of volumetric defects

Figure 2(c, d) and Table 1 indicate that the LoFs, GEPs, and KHs all have some degrees of overlap in size (i.e., max. axis), aspect ratio, and sphericity. In P^+20%^V^−40%^ (see Fig. 2(d)), both KHs and GEPs are observed at the aspect ratio of 0.8, and both LoFs and KHs are found to have a sphericity of 0.8. An overlapping nature refers to the tendency of having a common measure of certain morphological parameters for the considered types of defects, which is perhaps the reason why existing criteria, thresholding one or two parameters, are insufficient to reliably classify the volumetric defects^21,23,24,34^. It naturally follows that a criterion utilizing multiple parameters should reduce the probability of misidentification. For instance, a 50% overlap in each parameter between two defect types only translates to 25% overlap (50% × 50%) when two parameters are utilized, and 12.5% (50%×50%×50%) for three parameters, and so on.

**Fig. 3:**
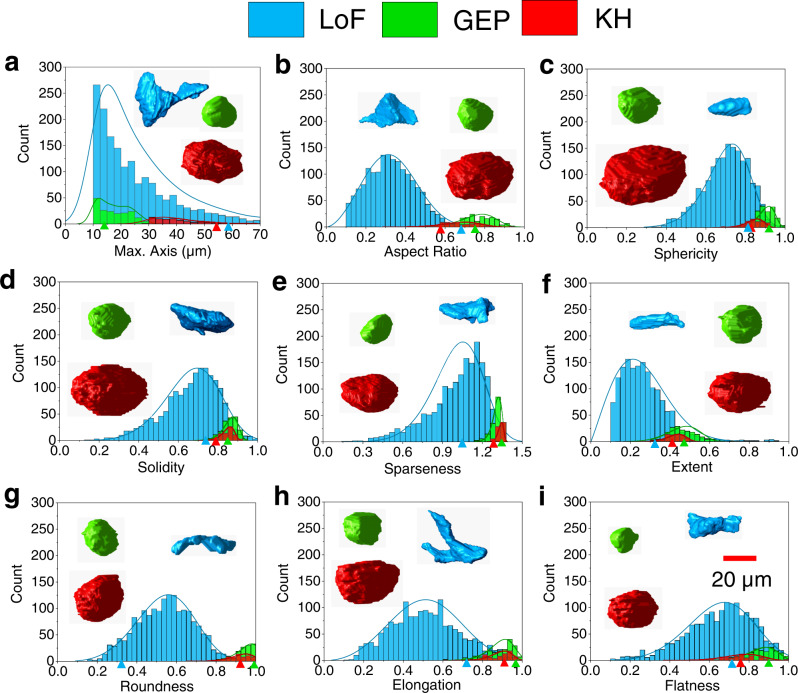
Distributions of morphological parameters for LoFs, GEPs, and KHs. Parameters include **a** max. axis, **b** aspect ratio, **c** sphericity, **d** solidity, **e** sparseness, **f** extent, **g** roundness, **h** elongation, and **i** flatness. Examples of each type of defects are also shown in the panels with the values of their morphological parameters pointed by triangles of respective colors. The fitted curves are Kernel Smooth for max. axis, while Weibull distributions are used for all other parameters.

Hence, six other morphological parameters are calculated in this study for each labeled defect to facilitate their classification. These parameters include solidity, sparseness, extent, roundness, elongation, and flatness (see Supplementary Table 2 and Supplementary Fig. 3 for definition and graphical representation of the parameters). These morphological parameters are derived from the understanding of defects and their potential impacts on L-PBF part fatigue performance, not only reducing the data dimension, but also increasing the interpretability of classification. In addition to the defects in P^−20%^V^0%^ and P^+20%^V^−40%^ coupons, defects observed in XCT scans of 20 other coupons fabricated using altered process parameters are also visually inspected, labeled, and their morphological parameters calculated (see Methods section for complete details on fabrication and labeling of defects). The distributions of all LoFs, GEPs, and KHs in each of the nine morphological parameters are presented and compared in Fig. 3. Similar to size, aspect ratio, and sphericity, overlaps also exist in the six additional parameters among different defect types. In addition, the degree of overlaps appears to depend on the defect type and the morphological parameter calculated. For instance, as shown in Fig. 3(d), while the solidity range of GEPs is completely contained within that of the LoFs, this overlapped range only accounts for ~35% of LoFs’ total range. In addition, unlike solidity, the roundness ranges of these two GEPs and LoFs only have a limited overlap (Fig. 3(g)).

**Fig. 4:**
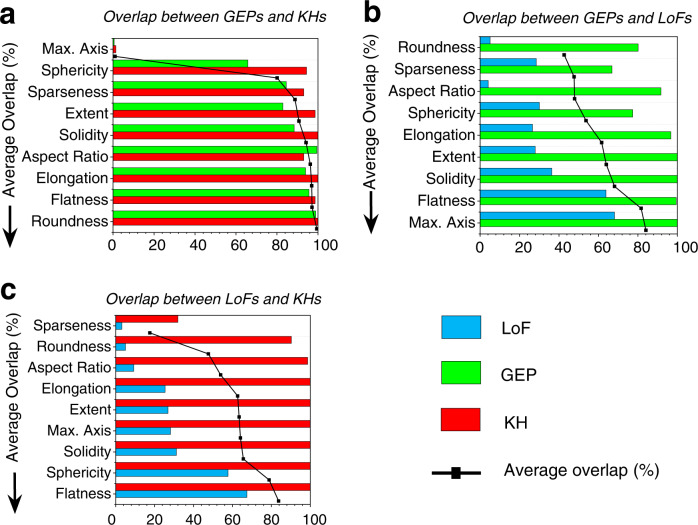
Pairwise ranking of the morphological parameters among LoFs, GEPs, and KHs. Bar charts showing the degrees of overlaps in the morphological parameters between **a** GEPs and KHs, **b** GEPs and LoFs, and **c** LoFs and KHs. Bars of each color represents the percentage that the overlapped ranges occupy the respective total ranges of each defect type.

The degrees of overlaps in all nine morphological parameters between each two of the three defect types have been quantified and presented in Fig. 4(a–c). A smaller overlap indicates a larger differentiating potential of a parameter. For instance, it is quite notable from Fig. 4(a), that the max. axis is the most distinguishing feature between KHs and GEPs, followed by sphericity which is significantly less effective. This is intuitive since both GEPs and KHs are quite spherical—with KHs being less so—but the size of the latter is significantly larger (Fig. 1). However, due to the large size range of LoFs (Fig. 3(a)) that covers the entire ranges of GEPs and KHs, the max. axis is no longer effective in differentiating GEPs from LoFs and KHs from LoFs. Instead, since LoFs shape tends to be flat and irregular (see examples given in Fig. 3), they are most effectively distinguished from GEPs and KHs by roundness and sparseness (see Fig. 4(b, c)). The sparseness ranges of KHs and LoFs are the least overlapped (Fig. 3(e)) in contrast to the roundness being the least for GEPs and LoFs (Fig. 3(g)). Counterintuitively, significant overlaps in sphericity between GEPs and LoFs and between KHs and LoFs are noticed in Fig. 3(c), which is primarily due to the existence of very small LoFs (max. axis 10–20 μm) which tend to have relatively high sphericity.

Based on the pairwise rankings presented in Fig. 4, a ranking of the morphological parameters’ overall effectiveness in distinguishing the three defect types can be generated. As shown in Table 2, the overall ranking starts by choosing the least overlapped parameter from the top of the pairwise lists, i.e., max. axis for GEPs and KHs (Fig. 4(a)), roundness for GEPs and LoFs (Fig. 4(b)), and sparseness for LoFs and KHs (Fig. 4(c)). The max. axis ranges of GEPs and KHs only contain 0.79% of total defects in their overlapped portions, which is far superior to the roundness of GEPs and LoFs, and sparseness of LoFs and KHs. Hence, max. axis has the Rank 1 (see Table 2). Afterward for Rank 2, having max. axis eliminated, sphericity from the pairwise rank of GEPs and KHs is compared with the roundness of GEPs and LoFs and sparseness of LoFs and KHs. This selects sparseness as the second most differentiating parameter. This process is continued until all nine parameters are ranked.

**Table 2:** Generating overall ranks for the morphological parameters considering all defect types (i.e., LoFs, GEPs, and KHs)

Rank	Percentage fraction of defects in the overlapped ranges			Selected parameter
	GEP & KH	KH & LoF	LoF & GEP	
1.	Max. Axis (0.79)	Sparseness (4.30)	Roundness (12.28)	Max. Axis
2.	Sphericity (73.91)	Sparseness (4.30)	Roundness (12.28)	Sparseness
3.	Sphericity (73.91)	Roundness (8.44)	Roundness (12.28)	Roundness
	Sphericity (73.91)	Aspect Ratio (12.86)	Roundness (12.28)	
4.	Sphericity (73.91)	Aspect Ratio (12.86)	Aspect Ratio (12.49)	Aspect Ratio
	Sphericity (73.91)	Aspect Ratio (12.86)	Sphericity (34.56)	
5.	Sphericity (73.91)	Elongation (28.41)	Sphericity (34.56)	Elongation
6.	Sphericity (73.91)	Extent (29.79)	Sphericity (34.56)	Extent
7.	Sphericity (73.91)	Solidity (33.99)	Sphericity (34.56)	Solidity
8.	Sphericity (73.91)	Sphericity (59.42)	Sphericity (34.56)	Sphericity
	Sphericity (73.91)	Sphericity (59.42)	Flatness (67.23)	
9.	Sphericity (73.91)	Flatness (68.75)	Flatness (67.23)	Flatness

### Defect classification using decision tree

**Fig. 5:**
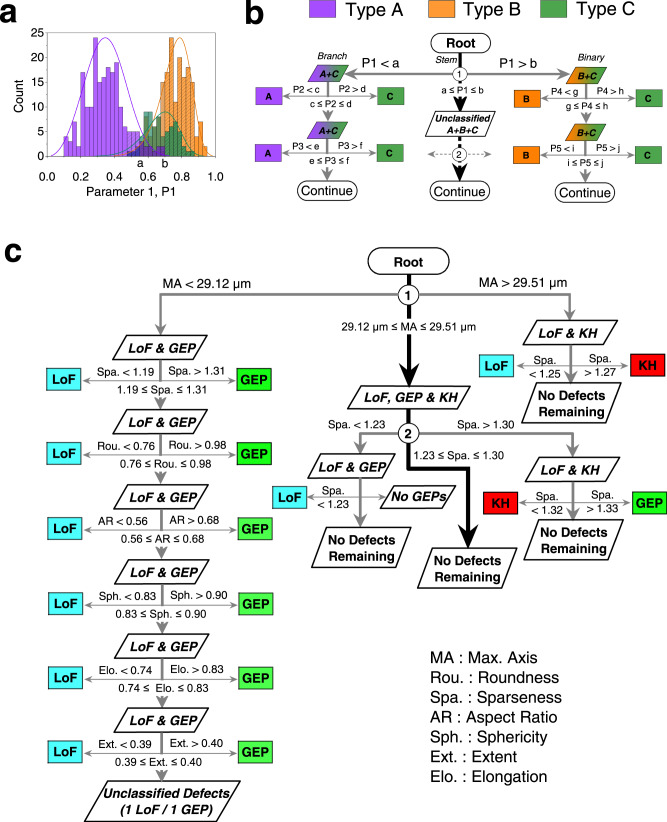
Decision tree for defect classification. **a** & **b** Schematic illustration of the methodology to generate the decision tree. **c** Decision tree generated based on the training data achieving an overall accuracy of 98.8% in the test data.

The facts that different degrees of overlap exist in the morphological parameters and that the most distinguishing parameter vary among different pairs of the three defect types suggest the effectiveness of a decision tree based on multiple morphological parameters to efficiently classify volumetric defects. Such a decision tree would “stem” from the most distinguishing parameter among the three pairs, “branches” at the boundaries of the overlapped ranges, and “grows” down the ranks of the parameters. As illustrated in Fig. 5(a, b), the main stem of the decision tree, which contains fully unclassified defects, splits at each node into binary branches (i.e., containing partially classified defects of two or fewer potential types). Starting from the “root”, the nodes on the stem sequentially select from the most distinguishing parameters, such as the list shown in Table 2. The stem grows until the end of the list or the exhaustion of fully unclassified defects. At each branch, depending on the potential types of defects contained, then traces down an appropriate binary list in Fig. 4(a–c) until the end of the list or the exhaustion of defects.

Following this approach, the decision tree is first validated with 5-fold cross-validation for its consistency in classification, and it achieves high classification accuracy of 97.9% with standard deviation of 0.76%. Then a decision tree has been generated based on the training data (i.e., randomly selected 70% of all the labeled defects, leaving 30% as test data), as shown in Fig. 5(c). Based on the training data, the distributions of the morphological parameters (similar to Fig. 3), bar charts showing the degree of overlaps between the defect types (similar to Fig. 4), and the overall ranking of the parameters considering all defect types (similar to Table 2) are provided in Supplementary Note 4. It is evident from Supplementary Table 3 that the top 6 parameters from the overall ranking based on the training dataset are identical to that of the complete dataset (Table 2). As shown in Fig. 5(c), the stem of the decision tree branches at Node 1 according to the boundaries of the overlapped range of max. axis—Rank 1 parameter in Supplementary Table 3—into two categories: GEPs+LoFs and KHs+LoFs. The binary branches (GEPs + LoFs and KHs+LoFs) then proceed down the respective ranks shown in Supplementary Figs. 5(b, c) until no more defects can be further classified. The stem of the tree containing unclassified defects would continue to branch at additional nodes according to lower-ranked parameters in Supplementary Table 3. However, after Node 2, no more fully unclassified defects remain. The so-generated decision tree, shown in Fig. 5(c), yields 98.8% accuracy when classifying the testing dataset (i.e., the remaining 30% of the labeled defects). Although the classification accuracy reported here is solely based on labeled data (unlabeled data were excluded), the model could potentially classify unlabeled data as well. However, the unlabeled defects classified by the models could not be verified as these defects could not be classified by human.

### Defect classification using an artificial neural network

The methodology employed in the decision tree approach of utilizing multiple morphological parameters—from the most discriminating to the least—to classify defects can also be implemented in artificial neural network (ANN). The ANN is a supervised ML technique that can establish the complex relationships between defect types and morphological parameters of the defects using layers of connected nonlinear modules called “neurons”^35^. In this work, using the morphological parameters of a defect as the input layer, the ANN model would assign its confidences (percent probability) in the defect being a GEP, LoF, or KH in the output layer. The defect would be assigned to the type with the highest confidence. The details regarding the construction of the ANN models, including Bayesian optimization of their architecture, data used, and the training procedure, are provided in Supplementary Note 6.

**Fig. 6:**
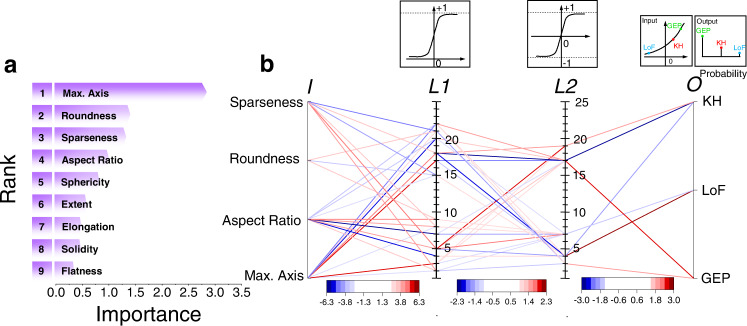
Artificial neural network for defect classification. **a** Rankings of the morphological parameters in terms of their importance obtained via PFI analysis. **b** Architecture of the ANN model: only connections with relatively large weights (i.e., large absolute values) in the ANN are shown. *I*, *L1*, *L2*, and *O* represent input layer, 1^st^ hidden layer, 2^nd^ hidden layer, and output layer of ANN, respectively.

It is notable that the accuracy of ANN models does not always improve with increasing morphological parameters. Instead, the best accuracy is typically achieved by using a selected few most discriminating parameters. Therefore, permutation feature importance (PFI) analysis can be used to first rank the discriminating potential among all the morphological parameters^36^. With an ANN constructed containing all morphological parameters in its input layer, the PFI analysis measures the “importance” of the morphological parameters by separately permutating the values of each morphological parameter and calculating the increase in the misclassification rate. A morphological parameter is deemed important if permutating its values results in a large increase in the misclassification rate. The rankings of the morphological parameters by the PFI analysis are shown in Fig. 6(a), highlighting their importance. Interestingly, the PFI ranking is similar to the one generated based on the relative data overlaps (the right column of Table 2)—in fact, the top four most distinguishing parameters in both rankings are identical.

According to the ranking generated by PFI analysis (Fig. 6(a)), a series of ANN models are constructed with an increasing number of morphological parameters used, which respectively results in classification accuracies of 98.3% (standard deviation: 0.33%), 98.8% (standard deviation: 0.29%) and 99.2% (standard deviation: 0.58%) in 5-fold cross-validations when the top two, three, and four most important morphological parameters are used. Including more parameters does not improve the accuracy of the ANN further. Then with the same split of 70%/30% of the labeled defects as in the decision tree, the essential architecture of the ANN model with four parameters in the input layer (top four from Fig. 6(a)) and classification accuracy of 99.0% on testing data is displayed in Fig. 6(b), only showing the connections among layers with relatively large weights. It includes two hidden layers of neurons, each of which is a nonlinear function taking the linear combinations of the previous layer’s output as its sole input variable. As shown in Fig. 6(b), the first layer contains 24 neurons of a logistics sigmoid activation function and the second layer contains 25 neurons of a hyperbolic tangent sigmoid activation function in the second layer. The output layer then uses a SoftMax transfer function to generate the probabilities of a defect being any of the three types.

Interpreting the nonlinear relationships between the input and output layers of ANN is generally difficult. Despite this, tracing neuronic connections with relatively large weights can shed some light qualitatively. For instance, in Fig. 6(b), both KH and GEP types have strong connections (being negative and positive, respectively) with neuron 17 in the 2nd hidden layer, which receives strong inputs from neurons 17, 18, and 22 in the 1st hidden layer—all of them are also strongly connected with max. axis. This indicates that the distinction between the KH and GEP types is more strongly influenced by max. axis, which is consistent with ranking based on the degree of overlapped ranges shown in Fig. 4(a). Similarly, Fig. 4(c) indicates that sparseness, roundness, and aspect ratio are the most distinguishing factor between KHs and LoFs. This is reflected in the connections in the ANN (Fig. 6(b)) noting that the LoF type is strongly connected to neuron 4 in the 2nd hidden layer, which connects strongly with both sparseness, roundness, and aspect ratio via neurons 18, 15, and 5 in the 1st hidden layer. On the other hand, KH type not only connects with max. axis as mentioned above, but it also connects with sparseness, roundness, and aspect ratio via neurons 17, and 4 in the 2nd hidden layer and neurons 18, 15, 5, and 4 in the 1st hidden layer. A very similar observation can be made in Fig. 6(b) regarding the connections from GEP and LoF to sparseness, roundness, and aspect ratio, which is consistent with the ranking shown in Fig. 4(b).

## Discussion

**Fig. 7:**
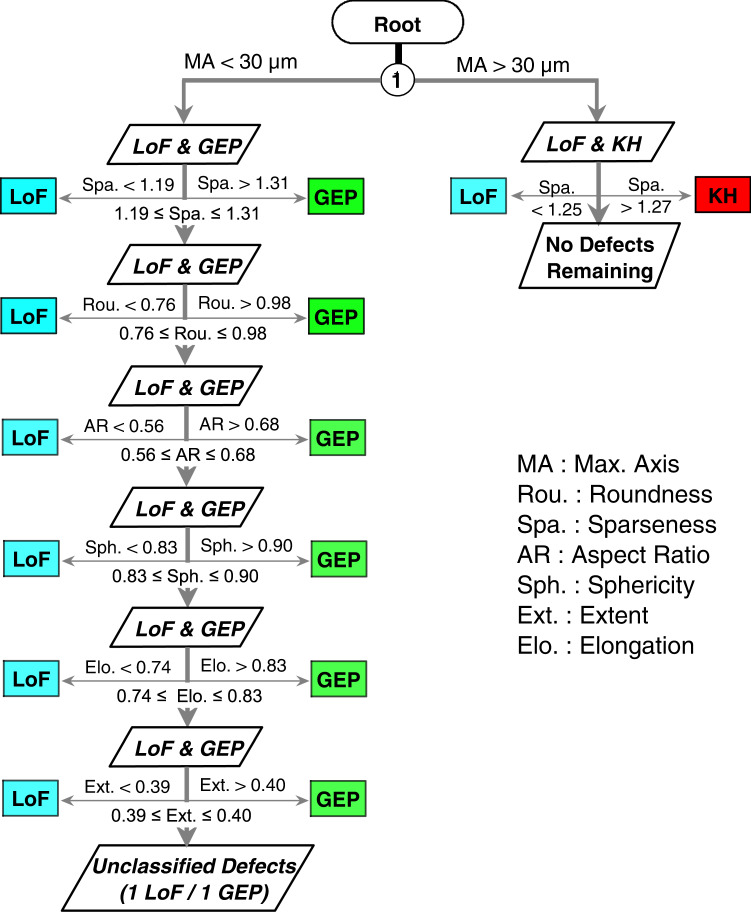
Decision tree for classification of defects in L-PBF Ti-6Al-4V. The simplified decision tree utilizing a 30 µm size threshold between GEPs and KHs during the classification of volumetric defects.

This work has demonstrated that volumetric defects in L-PBF Ti-6Al-4V, as identified from high-resolution XCT scans, can be classified into KHs, GEPs, and LoFs at great accuracy by simultaneously utilizing several morphological parameters which have relatively small overlaps among the respective ranges of the three defect types. In fact, Fig. 3(a) illustrates a very limited overlap between the max. axis ranges for GEPs and KHs, which is echoed by the observations made from the neural network (Fig. 6(b)) and suggests that a simple limit (e.g., at 30 µm) imposed on this parameter may be effective to distinguish the two defect types in L-PBF Ti-6Al-4V. In fact, all but one KH have size over 30 µm and all GEPs are smaller than 30 µm. As a result, the decision tree shown in Fig. 5(c) can be further simplified by ignoring the small overlap of max. axis at Node 1, see Fig. 7. The accuracy of this simplified decision tree is also quite high, reaching 98.8%.

During the construction of both decision tree and ANN models, it has been found that including more parameters does not always increase the accuracy of the models. In fact, the accuracy of the decision tree approach does not increase after including the seven most distinguishing parameters (see Fig. 5) and the ANN approach does not gain further accuracy after including the fourth parameter. In either case, extent and solidity do not improve the classification accuracy even though both are similar measures to sparseness (second most discriminating) and compare the volume of a defect with that of an enclosing geometry (see Supplementary Fig. 3 and Supplementary Table 2). The sparseness, extent, and solidity compare the volume of the defect with a fitted ellipsoid, a bounding box, and a convex hull, respectively. The superiority of the sparseness may be due to the ellipsoids’ resemblance to GEPs and KHs and their dissimilarity to LoFs. In contrast, such resemblance does not exist for bounding boxes or convex hulls.

Leveraging ML methods to investigate defect features of different defect types from XCT requires understanding different ML methods^37^. In general, high-resolution XCT images can improve the classification accuracy of ML methods. Incorporating the knowledge of defects and their types into the data-driven analysis can help the selection of appropriate ML methods and improve their accuracy and interpretability. In this study, with a relatively small amount of data, decision trees and ANN can model the nonlinear relationships between defect features and defect types, achieving high classification accuracy and sufficient interpretability. In comparison, other conventional ML methods (e.g., random forest, support vector machine, Gaussian process) are difficult to interpret with complex model structures; regression-based classification methods (e.g., logistic regression) can only model the linear relationship inadequate for our data; deep learning methods (e.g., deep neural network, convolutional neural network) need a huge amount of data in intensive training to achieve highly accurate but hardly interpretable results.

The morphological characteristics of defects depend on the material, fabrication technology, and resolutions of the XCT scans. For instance, the variations in thermal-physical properties among materials such as thermal conductivity, specific heat, surface tension, etc. influence the melt pool dynamics^38^ and can affect the size and shape of defects of each type. Similarly, defect characteristics are also influenced by feedstock, delivery method, heat source, and scan strategy which can vary from one AM technology to another^12,13,39–41^. Therefore, the same variations in process parameters performed in this work may result in different defect contents in other materials, processes, and even the same material/process but using a feedstock with different characteristics. In such cases, the proposed methodology may need to be re-calibrated.

Lastly, the voxel size used during the XCT scans directly influences the level of details of volumetric defects that can be captured. With larger voxel sizes, scans can miss certain features of defects, such as fine “ribs” on the KH surfaces, misidentify a larger LoF defect as a few smaller ones, or leave smaller defects undetected altogether^32,42–44^. As a result, the morphological parameters extracted from each defect and their value ranges for each defect type obtained at low-resolution may significantly differ from those at high-resolution, leading to higher probability of defects mislabeling. For these reasons, the statistics of defects’ morphological parameters, decision tree, and the ANN model reported in this work are specific to L-PBF Ti-6Al-4V coupons, XCT scanned at the voxel size of 1 µm (which offer a proper balance between resolution and scan time). With the low-fidelity data labeled from low-resolution scans, the efficacy of the methodology put forth by this work may naturally reduce. Nevertheless, this approach; i.e., leveraging multiple discriminating morphological parameters for defect classification, is expected to deliver significantly better classification accuracy compared to threshold-based methods and be generally applicable to any material, process, and XCT scan parameters.

## Methods

### Material and fabrication procedure

**Fig. 8:**
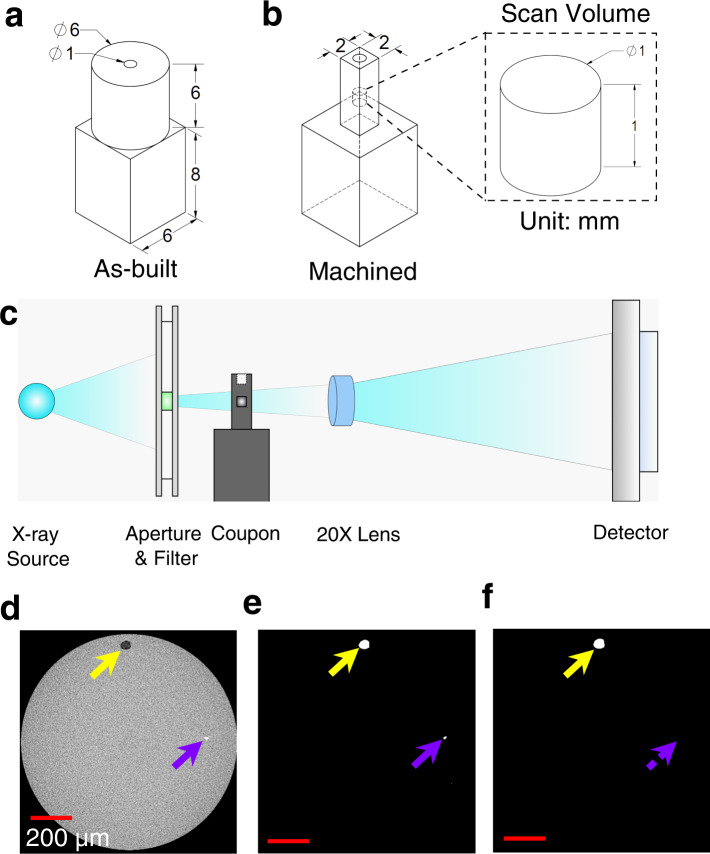
Experimental setup including geometry of the coupons and the XCT scans. Geometry and dimension of **a** as-built and **b** machined XCT coupon showing the scan volume of diameter and height of 1 mm. **c** Schematic illustration showing the XCT setup for Ti-6Al-4V coupons. Post-processing of XCT data: **d** volumetric scan image, **e** corresponding binary output, and **f** binary output excluding the high-density phase. The scale bars in (**d**–**f**) represent 200 µm. As a note, solid yellow and purple arrows point to a defect and a high-density phase, respectively. In addition, the dotted purple arrow signifies the removal of high-density phase from the analysis.

Plasma atomized Ti-6Al-4V Grade 5 powder (particle size range of 15 to 53 µm) supplied by AP&C – a GE Additive company was used for coupon fabrication in an EOS M290 machine (L-PBF method). During fabrication, the process parameters were altered from the manufacturer’s recommended values to induce different types of volumetric defects. The EOS recommended infill process parameters (i.e., 280 W laser power, 1300 mm/sec laser speed, 40 µm layer thickness, 120 µm hatch distance, 67° inter-layer rotation, and 10 mm stripe width) for Ti-6Al-4V Grade 5 material was used. Two sets of coupons were fabricated. The first set was fabricated by changing laser power and laser speed, and the second by changing laser power and hatch distance. Each set contained 49 coupons corresponding to ± 30% adjustment in two parameters at 10% intervals. The EOS “skywriting” and “time homogenization” features that allow uniform energy input throughout the cross-section of the coupons were enabled to avoid overheating and potential KH formation adjacent to the locations of laser turning^45^. As shown in Fig. 8(a), the coupons featured a cylindrical portion for XCT scanning and a square portion for gripping. The cylindrical portions of all coupons were later machined into rectangular bars of 2 mm thickness (see Fig. 8(b)) to permit high-resolution XCT scans in the infill region.

### X-ray computed tomography and labeling of volumetric defects

XCT scans were performed using a ZEISS Xradia 620 Versa machine. To reveal as much as possible the morphological features of the induced volumetric defects, the distance of the X-ray source and detector with respect to the coupon was arranged such that the scanned volumes were a cylinder with a diameter and height of 1 mm (see Fig. 8(b, c)), which corresponded to an isotropic voxel size of 1 µm. The scans were performed using an X-ray source of 160 kV voltage and 25 W power passing through a ZEISS “HE1” filter to remove the low-energy photons through the coupons^46^. These settings resulted in the photon transmittance of 20–25%. In addition, in each scan, 1601 2D projections were collected over a full 360 degrees rotation of the coupons.

After the completion of the scan, the volumetric tomography data were reconstructed using the ZEISS Reconstruction software. The reconstructed images were post-processed using Dragonfly Pro and ImageJ softwares^47,48^ to obtain the binary images as well as to remove the high-density phases (see Fig. 8(d–f)). The resulting binary output file (i.e., Fig. 8(f)) was further analyzed using MATLAB software to isolate the volumetric defects and calculate their morphological parameters. To minimize false defect identification, only defects with max. axis greater than 10 µm were included in the analysis.

Each volumetric defect was classified via human visual inspection into one of the three defect types; viz., LoF, GEP, and KH, by five individuals with experience in L-PBF processing and materials, and their typical defects. For labeling, the only biases the students had were their formation mechanisms and the general characteristics of their shape and size as was summarized in the introduction. After labeling, only those defects with the agreement of at least 4 out of 5 students were admitted for further analysis, the rest were rejected as inclusive. Out of 2156 total defects, the labeling of only 1970 defects were conclusive. Among these, 1717 were LoFs, 181 were GEPs, and 72 were KHs. The labeled defects with high confidence were only used for constructing training data, which was an essential step for obtaining reliable classification models. The more accurate the labelled defects in the training data, the more different patterns can be discovered, and more confident the models to classify both the defects in the labeled testing data and new defects. The 186 unlabeled defects were relatively small (smaller than 30 µm) with low criticality on fatigue strength, and they were not important to include in the analysis.

## Supplementary information

Supplementary Information

## Data Availability

The XCT data are available under restricted access for having large size in the order of TBs, which cannot be stored or transferred on commonly available data sharing platforms, access can be obtained by requesting from the corresponding author.

## Code availability

Defects were extracted from XCT scans using ImageJ and Dragonfly Pro. Figures were generated using Origin Pro and Microsoft PowerPoint. Analysis of results was performed by MATLAB with in-house developed codes, which can be accessed at 10.5281/zenodo.6869455.
