# Supplementary material for: Feature-based volumetric defect classification in metal additive manufacturing
Source: Nat Commun. 2022 Oct 26;13:6369. doi: 10.1038/s41467-022-34122-x (PMC9606371; doi:10.1038/s41467-022-34122-x)
Supplement: Supplementary file 1 — Supplementary Information [file 41467_2022_34122_MOESM1_ESM.pdf]

Supplementary Information for

Feature-based volumetric defect classification in metal additive manufacturing

Arun Poudel[1,2], Mohammad Salman Yasin[1,2], Jiafeng Ye[3], Jia Liu[3], Aleksandr Vinel[3], Shuai Shao[1,2], and Nima Shamsaei[1,2*]

[1]National Center for Additive Manufacturing Excellence (NCAME), Auburn University, Auburn, AL 36849, USA.

[2]Department of Mechanical Engineering, Auburn University, Auburn, AL 36849, USA.

[3]Department of Industrial and Systems Engineering, Auburn University, Auburn, AL 36849, USA.

*Email: shamsaei@auburn.edu

Supplementary Note 1. Overlapping nature of size, sphericity, and aspect ratio of volumetric defects seen in $P^{-20\%}V^{0\%}$ and $P^{+20\%}V^{-40\%}$ coupons.

The coupons fabricated using $P^{-20\%}V^{0\%}$ and $P^{+20\%}V^{-40\%}$ conditions (see also **Fig. 2** of the main text) consist of defects with varying morphology. The coupon fabricated in underheating conditions (i.e., $P^{-20\%}V^{0\%}$) mainly consists of lack of fusions (LoFs) with lower sphericity and aspect ratio and a small number of gas-entrapped pores (GEPs) (see **Supplementary Fig. 1(a-c)**). The morphological characteristics seen in these figures are attributed to the flat and irregular shape of LoFs and the near-spherical shape of GEPs. On the other hand, the overheated coupon (i.e., $P^{+20\%}V^{-40\%}$) consists of all three types of defects (i.e., LoFs, GEPs, and keyholes (KHs)) (see **Supplementary Fig. 1(d-f)**). In general, GEPs are smaller with higher sphericity and aspect ratio as compared to LoFs and KHs. Furthermore, KHs have higher sphericity and aspect ratio as compared to LoFs but lower than GEPs. In both coupons, GEPs are smaller than ~30 µm. The ranges of size, sphericity, and aspect ratio of different types of defects appear to overlap one another to varying degrees.

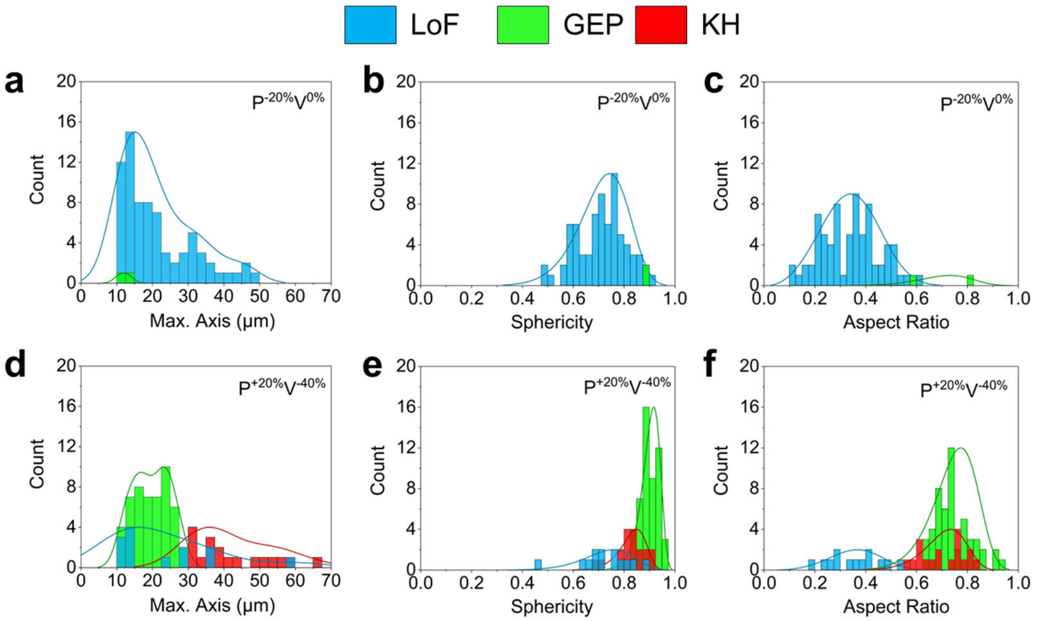

Supplementary Fig. 1: Distributions of morphological parameters for LoFs, GEPs, and KHs. Parameters include max. axis, sphericity, and aspect ratio of volumetric defects observed in coupons fabricated using **a-c** $P^{-20\%}V^{0\%}$ and **d-f** $P^{+20\%}V^{-40\%}$ parameters.

Supplementary Note 2. Evaluation of the existing classification criteria in the literature.

The classification criteria from the literature may have been developed for specific processes, fabrication conditions, and characterization techniques. As a result, they can only partially classify the volumetric defects observed in $P^{+20\%}V^{-40\%}$ and $P^{-20\%}V^{0\%}$ Ti-6Al-4V coupons (see **Supplementary Figs. 2**). The criteria proposed by Snell et al.[1] defines the size of LoFs and KHs to be greater than 100 μm; however, all the LoFs and KHs observed in the $P^{+20\%}V^{-40\%}$ and $P^{-20\%}V^{0\%}$ coupons are smaller than 100 μm. The criteria proposed by Kasperovich et al.[2] defines sphericity for LoFs to be smaller than 0.7; however, LoFs with sphericity as high as 0.9 are also observed in this work. The criteria proposed by Vilaro et al.[3] cannot classify the LoFs smaller than 100 μm. **Supplementary Fig. 2** only contains defect information from the $P^{+20\%}V^{-40\%}$ and $P^{-20\%}V^{0\%}$ coupons. A thorough evaluation of the existing criteria from the literature has also been performed with all defects in this study containing 1717 LoFs, 181 GEPs, and 72 KHs. The resulting numbers of accurately classified as well as misclassified defects are presented in **Supplementary Table 1.**

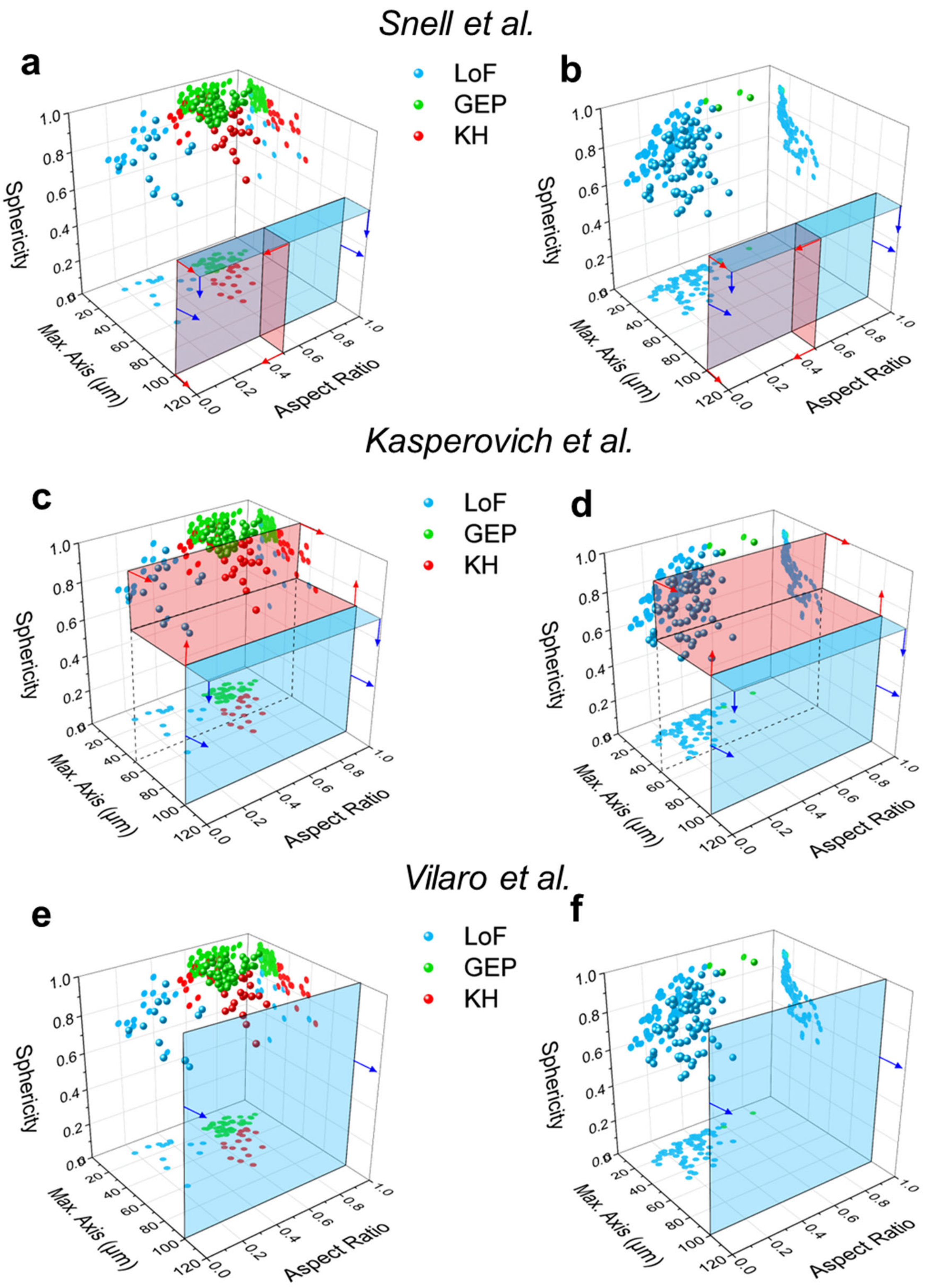

Supplementary Fig. 2: Evaluation of existing defect classification criteria. The criteria proposed by (**a** & **b**) Snell et al.[1], (**c** & **d**) Kasperovich et al.[2], and (**e** & **f**) Vilaro et al.[3] were evaluated using $P^{+20\%}V^{-40\%}$ and $P^{-20\%}V^{0\%}$ Ti-6Al-4V coupons, respectively.

Supplementary Table 1 Numbers of accurately classified and misclassified defects with the existing classification criteria from the literature. (Total dataset: 1717 LoFs, 181 GEPs, and 72 KHs)

Study	Defects	Criteria	No. of accurately classified defects	No. of misclassified defects
Snell et al.[1]	LoF	Size > 100 µm<br>Sphericity < 0.6	16	1701(LoF)
	KH	Size > 100 µm<br>Aspect ratio < 0.5	0	16(LoF)+72 (KH)
Kasperovich et al.[2]	KH	Size > 50 µm<br>Sphericity > 0.7	9	63(KH)+2(LoF)
	LoF	Size > 100 µm<br>Sphericity < 0.7	16	1701(LoF)
Vilaro et al.[3]	LoF	Size > 100 µm	16	1701(LoF)

Supplementary Note 3. Definitions of considered morphological parameters of defects.

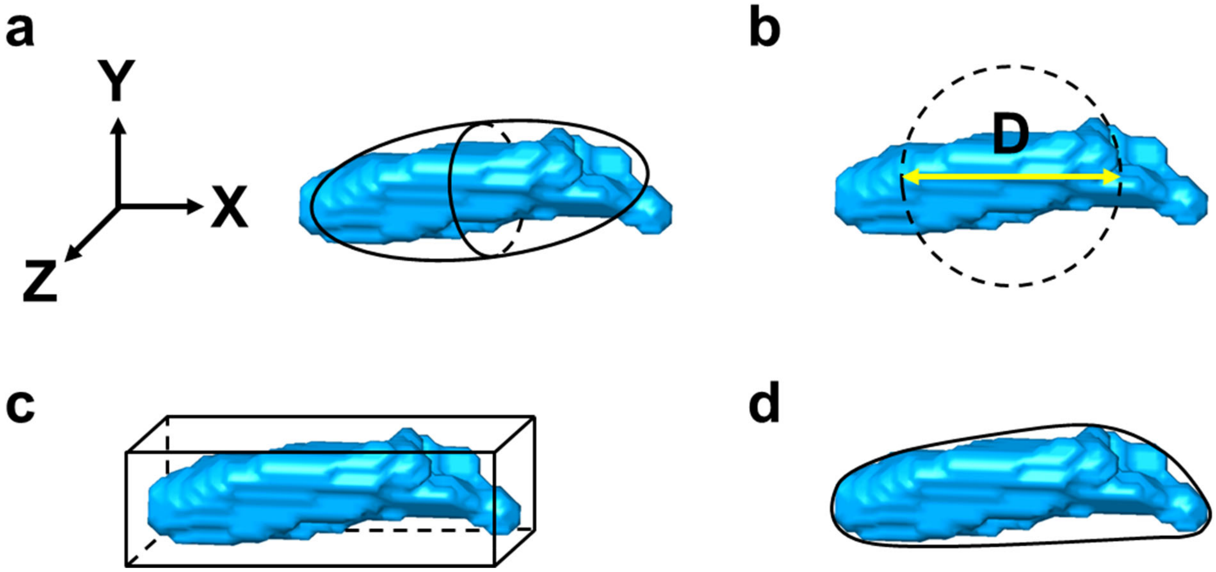

Supplementary Fig. 3: Graphical representation. **a** Fitted ellipsoid. **b** Equivalent diameter (D). **c** Bounding box. **d** Convex hull.

Supplementary Table 2 Definitions of considered morphological parameters of the defects.

	Defect parameters	Definitions
1.	Maximum axis	Length of the major axis of the fitted ellipsoid (see Supplementary Fig. 3(a)).
2.	Sphericity	Degree to which the shape of an object approaches the sphere. $$\text{Sphericity} = \frac{\pi^{1/3}\,(6V)^{2/3}}{A} \quad (1)$$ where V and A are the volume and curved surface area of the object, respectively.
3.	Aspect ratio	Ratio of minor to major axis of the fitted ellipsoid.
4.	Roundness[4]	Ratio of equivalent diameter (i.e., diameter of the sphere with the same volume as the defect (see Supplementary Fig. 3(b))) of the defect to its maximum axis.
5.	Extent	Ratio of volume of the object to the volume of the bounding box (see Supplementary Fig. 3(c)).
6.	Solidity	Ratio of volume of the object to that of convex hull surrounding it (see Supplementary Fig. 3(d)).
7.	Sparseness	Ratio of volume of the object to the volume of the fitted ellipsoid.
8.	Elongation	Ratio of median to major axis of the fitted ellipsoid.
9.	Flatness	Ratio of minor to median axis of the fitted ellipsoid.

Supplementary Note 4. Statistics of the training dataset used for decision tree and neural network.

The labeled defects with high confidence are used for constructing training data, which is essential for obtaining reliable classification models. The more accurate the labelled defects in the training data, the more different patterns can be discovered, and more confident the models to classify both the defects in the labeled testing data and new defects. Five-fold cross-validation is used to validate the consistency in the classification of both decision trees and neural networks on the labeled defects. Then, a randomly selected subset; i.e., 70% of all manually labeled volumetric defects, is used for the training of the decision tree and neural network. The statistical distributions of the morphological parameters of the defects in this subset are observed to be similar to the entire labeled defects (see **Supplementary Fig. 4** and **Fig. 3**). The ranges of parameters for each defect type are similar for both datasets. As a result, the percentage overlap observed for each defect type seen in **Supplementary Fig. 5** as well as the ranking of the parameters are consistent with the observations made in **Fig. 4**. **Supplementary Fig. 5(b)** indicates that the average percent overlaps for both roundness and sparseness of GEPs and LoFs are similar. This is also consistent with **Fig. 4(b)**, despite the difference in the ranks. Finally, following the methodology detailed in Section 3.2 of the main text, the morphological parameters are ranked for all defect types based on the training data in **Supplementary Table 3**.

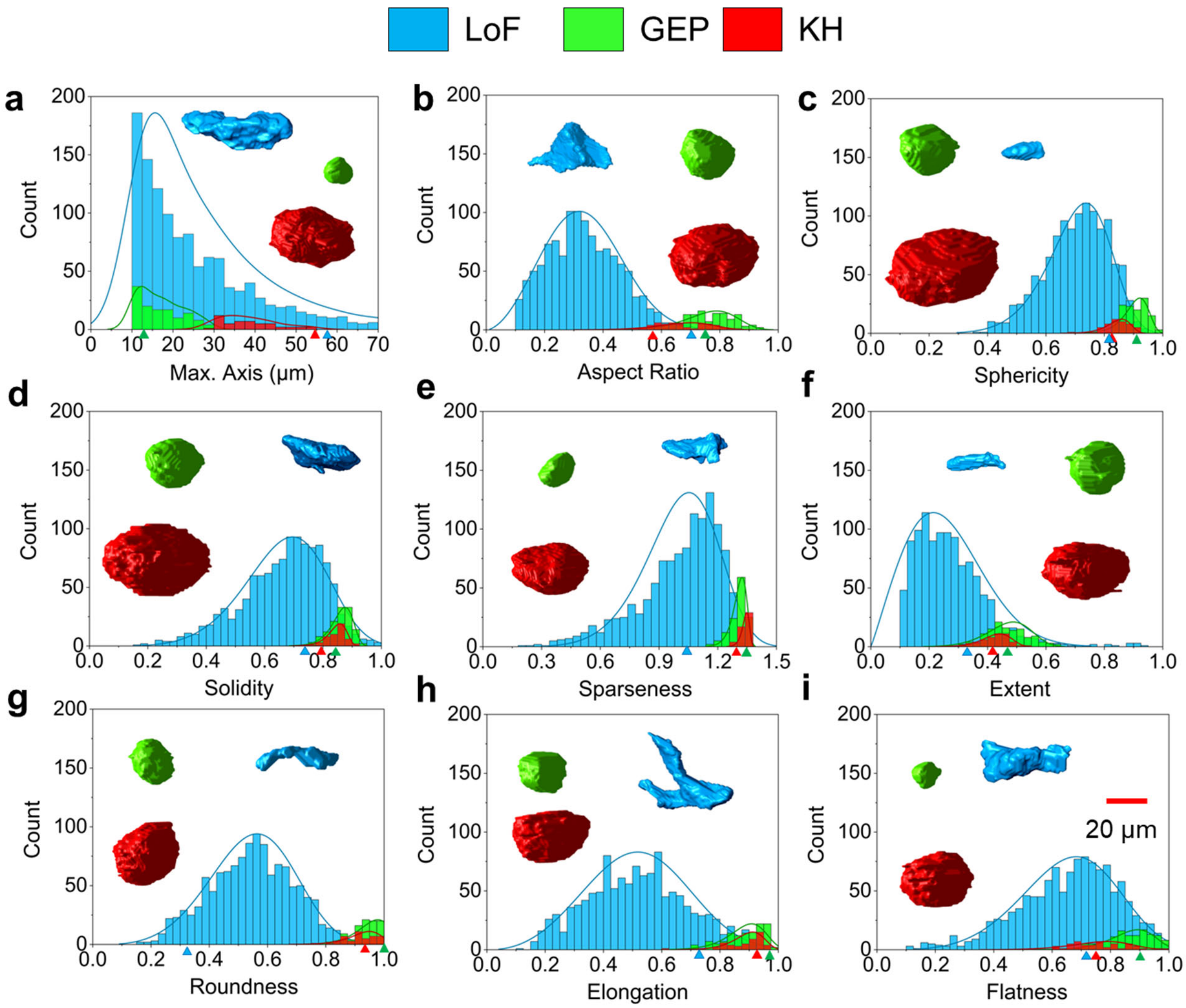

Supplementary Fig. 4: Distributions of morphological parameters for LoFs, GEPs, and KHs in training data—70% of the randomly selected defects. Parameters include **a** max. axis, **b** aspect ratio, **c** sphericity, **d** solidity, **e** sparseness, **f** extent, **g** roundness, **h** elongation, and **i** flatness. Examples of each type of defect are also shown in the panels with the values of their morphological parameters pointed by triangles of respective colors. The fitted curves are Kernel Smooth for max. axis, while Weibull distributions are used for all other parameters.

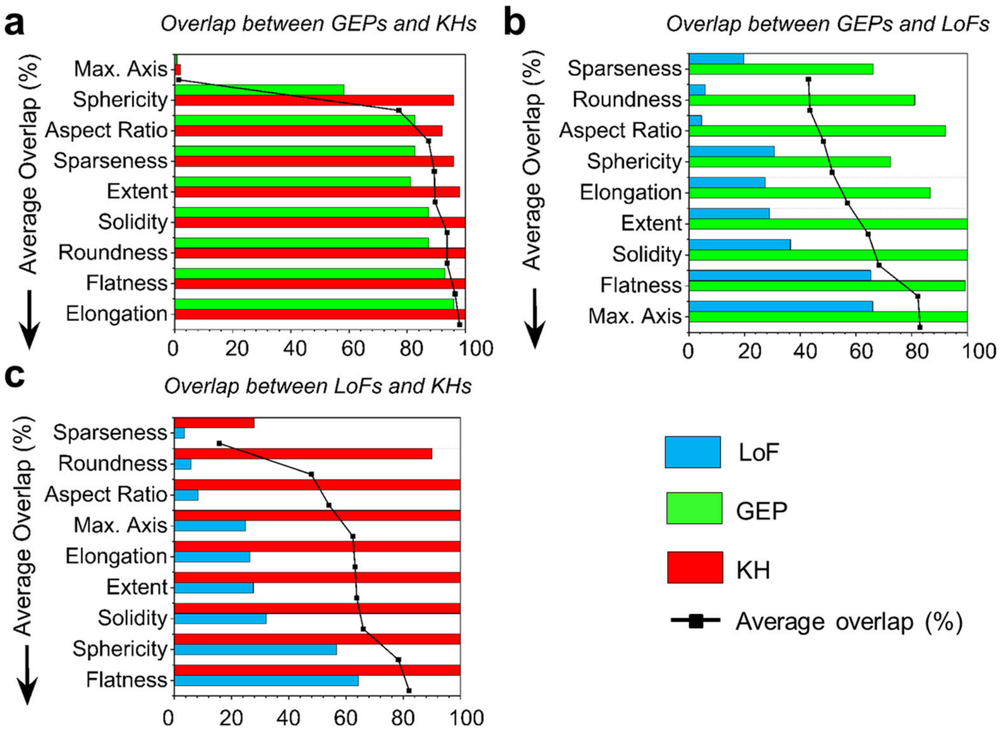

Supplementary Fig. 5: Pairwise ranking of the morphological parameters among LoFs, GEPs, and KHs in the training data. Bar charts showing the degrees of overlaps in the morphological parameters of defects in the training data between **a** GEPs and KHs, **b** GEPs and LoFs, and **c** LoFs and KHs. Bars of each color represent the percentage that the overlapped ranges occupy the respective total ranges of each defect type.

Supplementary Table 3 Generating overall ranks for the morphological parameters considering all defect types (i.e., LoFs, GEPs, and KHs). Only training data is used.

Node	Percentage Fraction of Defects in the Overlapped Ranges			Selected Parameter
	GEP & KH	KH & LoF	LoF & GEP	
1.	Max. Axis (1.13)	Sparseness (4.56)	Sparseness (24.17)	Max. Axis
2.	Sphericity (68.93)	Sparseness (4.56)	Sparseness (24.17)	Sparseness
3.	Sphericity (68.93)	Roundness (9.12)	Sparseness (24.17)	Roundness
4.	Sphericity (68.93)	Aspect Ratio (11.91)	Sparseness (24.17)	Aspect Ratio
	Sphericity (68.93)	Elongation (29.33)	Sparseness (24.17)	
5.	Sphericity (68.93)	Elongation (29.33)	Sphericity (34.64)	Elongation
6.	Sphericity (68.93)	Extent (30.53)	Sphericity (34.64)	Extent
7.	Sphericity (68.93)	Solidity (34.85)	Sphericity (34.64)	Sphericity
8.	Sphericity (68.93)	Solidity (34.85)	Solidity (42.55)	Solidity
	Sphericity (68.93)	Sphericity (58.43)	Solidity (42.55)	
	Sphericity (68.93)	Sphericity (58.43)	Flatness (68.52)	
9.	Sphericity (68.93)	Flatness (65.71)	Flatness (68.52)	Flatness

Supplementary Note 5. Automated decision tree model.

The machine learning (ML) based decision tree model uses a scoring criterion, namely Gini's impurity index[5], to create feature-based binary splits within the dataset iteratively. The impurity index illustrates the likelihood of wrongly classifying a certain type of defect in a random fashion, and is expressed as $G = 1 - \sum p_i^2$, where p_i is defined by defect belonging to a certain class at any given node. The root node branches iteratively using the low impurity index value found from different parameters and with minimum branch node having at least 10 defects.

The original dataset has 1717 LoFs, 181 GEPs, and 72 KHs. The automated decision tree is also first validated with 5-fold cross-validation and it achieves high classification accuracy of 98.0% with a standard deviation of 1.19%. Then, the dataset has been divided into two groups in which 70% of the data are randomly selected for training and the remaining are reserved to test the accuracy of the model. All nine morphological parameters are used as the input variables, whereas the causative variable contains the type of defects (i.e., LoF, KH, or GEP). The result from the ML based decision tree model is presented in **Supplementary Fig. 6** and an accuracy of 98.1% can be achieved during defect classification.

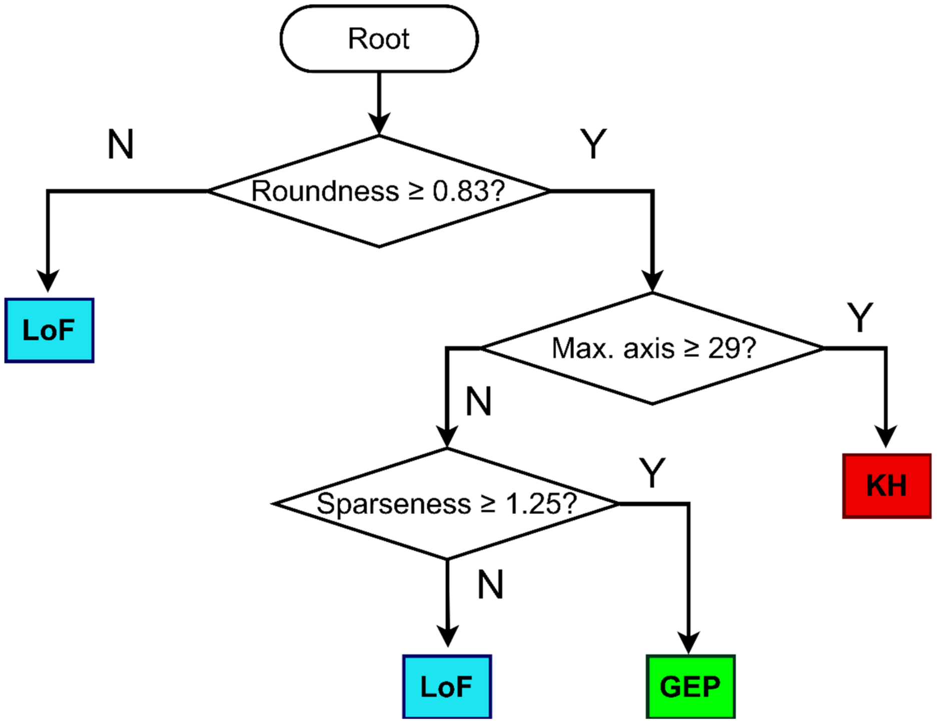

Supplementary Fig. 6: Automatedly generated decision tree for classification of LoF, GEP, and KH defects. Overall accuracy of 98.1% was observed.

Supplementary Note 6. Artificial neural network model.

An artificial neural network (ANN) aims to model complex nonlinear relationships between predictors and the response in the dataset by using an architecture consisting of an input layer, hidden layers, and an output layer with basic nonlinear transformation units called "neurons" [6]. In ANN, for the jth neuron on the kth hidden layer, the nonlinear transformation is achieved by using Equation (2).

$$x_j^k = \sigma\left(\sum_{i=1}^{I} W_{ij}^k x_i^{k-1} + b_j\right), j = 1, \dots, J, k = 1, \dots, K \qquad (2)$$

where x_j^k is the output of the jth neuron on the kth hidden layer, x_i^{k-1} is the output of the ith neuron on the previous layer. I and J are the number of neurons on the two consecutive layers, K is the total number of hidden layers. They are the hyperparameters of the ANN model, and can be optimized during model training to improve model accuracy. Moreover, W_{ij}^k is the weight connecting the ith neuron on the $(k-1)$th layer to the jth neuron on the kth layer, b_j is the bias

for the kth layer, and they are updated iteratively during the training process. $\sigma(\cdot)$ is a nonlinear activation function (e.g., the logistic sigmoid function). The predictors x_i^0 $(i = 1, \dots, I)$ is used as input for the neurons on the input layer. For classification, the neurons on the output layer usually use a SoftMax transfer function (see Equation (3)) to generate the probabilities of response y for different classes $(c = 1, \dots, C)$.

$$\text{Softmax}\left(\exp\left(\sum_{j=1}^{J} W_{jc}^{K+1} x_j^K\right)\right)_c = \frac{\exp\left(\sum_{j=1}^{J} W_{jc}^{K+1} x_j^K\right)}{\sum_{c=1}^{C} \exp\left(\sum_{j=1}^{J} W_{jc}^{K+1} x_j^K\right)} \coloneqq P(y = c) \tag{3}$$

where x_j^K is the output of the jth neuron on the last hidden layer (Kth), W_{jc}^{K+1} is the weight connecting the jth neuron on the last hidden layer and the cth neuron (representing class c) on the output layer, and $P(y = c)$ is the probability of labeling the response y as class c. ANN usually classifies the response into the class with the highest probability.

In our case study, ANN classifies defects into three classes (i.e., KH, LoF, and GEP) represented by three neurons on the output layer, from various predictors (i.e., defect features, like max axis length, aspect ratio, sphericity, and roundness) represented by four neurons on the input layers. Two hidden layers with 24 neurons and 25 neurons, respectively, are identified in the ANN model. After training, the ANN model can yield a high classification accuracy of 99.0% on new defects in a testing dataset.

Supplementary References

1. Snell, R. *et al.* Methods for Rapid Pore Classification in Metal Additive Manufacturing. *J. Miner. Met. Mater.* **72**, 101–109 (2020).

2. Kasperovich, G., Gussone, J., Haubrich, J., Schulte, D., Requena, G. Correlation between porosity and processing parameters in TiAl6V4 produced by selective laser melting. *Mater. Des.* **105**, 160–170 (2016).

3. Vilaro, T., Colin, C. & Bartout, J. D. As-fabricated and heat-treated microstructures of the Ti-6Al-4V alloy processed by selective laser melting. *Metall. Mater. Trans. A Phys. Metall. Mater. Sci.* **42**, 3190–3199 (2011).

4. Garczyk, Ż. & Stach, S. Three-dimensional model for assessing the pore volume of biomaterials intended for implantation. *Comput. Model. Biomech. Biotribology Musculoskelet. Syst.* 305–358 (2021) doi:10.1016/B978-0-12-819531-4.00013-4.

5. Myles, A. J., Feudale, R. N., Liu, Y., Woody, N. A. & Brown, S. D. An introduction to decision tree modeling. *J. Chemom.* **18**, 275–285 (2004).

6. Wang, S.-C. Artificial Neural Network. *Interdiscip. Comput. Java Program.* 81–100 (2003).
